# Emerging Non-Thermal Technologies for the Extraction of Grape Anthocyanins

**DOI:** 10.3390/antiox10121863

**Published:** 2021-11-23

**Authors:** Antonio Morata, Carlos Escott, Iris Loira, Carmen López, Felipe Palomero, Carmen González

**Affiliations:** enotecUPM, Chemistry and Food Technology Department, Technical University of Madrid UPM, 28040 Madrid, Spain; carlos.escott@gmail.com (C.E.); iris.loira@upm.es (I.L.); carmen.lopez@upm.es (C.L.); felipe.palomero@upm.es (F.P.); carmen.gchamorro@upm.es (C.G.)

**Keywords:** non-thermal technologies, grapes, wine, anthocyanins, HHP, UHPH, PEFs, US, irradiation

## Abstract

Anthocyanins are flavonoid pigments broadly distributed in plants with great potential to be used as food colorants due to their range of colors, innocuous nature, and positive impact on human health. However, these molecules are unstable and affected by pH changes, oxidation and high temperatures, making it very important to extract them using gentle non-thermal technologies. The use of emerging non-thermal techniques such as High Hydrostatic Pressure (HHP), Ultra High Pressure Homogenization (UHPH), Pulsed Electric Fields (PEFs), Ultrasound (US), irradiation, and Pulsed Light (PL) is currently increasing for many applications in food technology. This article reviews their application, features, advantages and drawbacks in the extraction of anthocyanins from grapes. It shows how extraction can be significantly increased with many of these techniques, while decreasing extraction times and maintaining antioxidant capacity.

## 1. Introduction

Anthocyanins are flavonoid pigments responsible for the color of many fruits, flowers and vegetable tissues. Extensive details on their properties and features can be found in the literature [1,2,3,4,5,6,7,8,9]. They have been extensively studied for their potential applications as natural colorants [10,11,12] as they are innocuous and safe molecules, but also for their positive impact on health due to their antioxidant properties [13,14] and their effect on the gut microbiome [15]. Anthocyanin color depends on the substitution pattern in the B-ring and the acylation patterns, both of which affect the electron density and the observed color, ranging in grapes from red orange (brownish red) to bluish red (purple), with typical ranges from 518 nm of maximum absorption for cyanidin to 528 nm for malvidin [16]. Acylation normally increases the maximum absorption (e.g., malvidin 528 nm to coumaroyl malvidin 535 nm) (Table 1). The color of anthocyanins is also affected by low pH, which increases the color intensity by the hyperchromic effect, shifting the equilibria to increase the amount of pyrilium cation. Additionally, anthocyanins can also undergo SO_2_ bleaching and co-pigmentation processes that produce bluish red pigments by bathochromic shifts in the maximum wavelength of absorbance [16].

**Figure 1:**
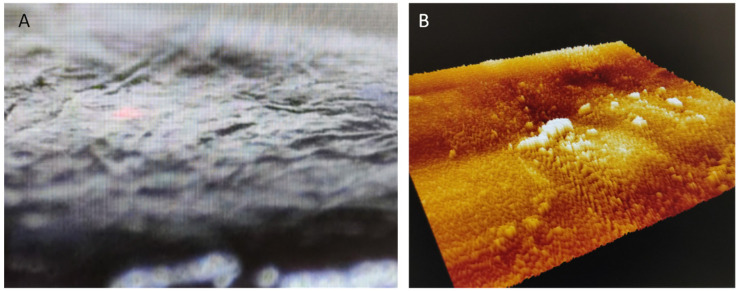
(**A**) Red grape skin (exocarp) *Vitis vinifera* L. Tempranillo variety by 60 µm optical camera built-in part of the AFM. (**B**) 3D Topography of the same skin by atomic force microscopy.

In most grape varieties, anthocyanins are located in the exocarp (skins) (Figure 1A,B), which are the layers of cells in the outer surface of the berry; only a few varieties also have anthocyanins in the pulp [16]. The skins have a thicker cell wall than the pulp to protect the berry mechanically and against rot and pests.

**Figure 2:**
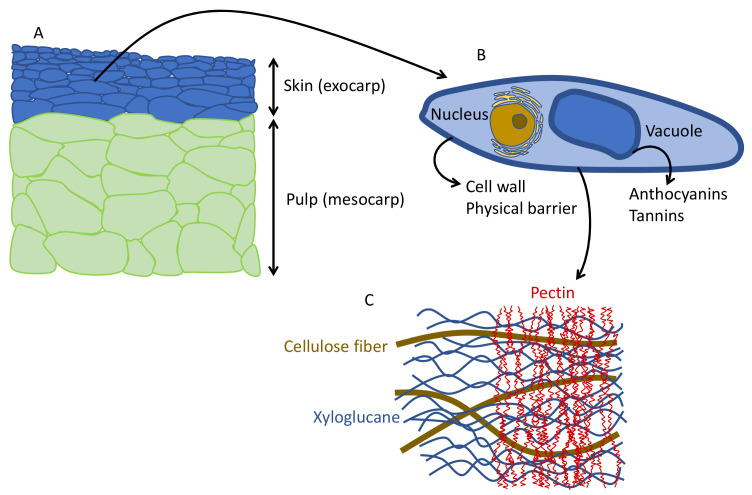
(**A**) Red grape section with flat colored cells in the skins and polyhedral cells in the pulp. (**B**) Skin cells shape and structure. (**C**) Cell wall fiber components.

The structure and shape of the cells in the berries are flat cells in the skin and large polyhedral cells in the pulp (Figure 2A). Anthocyanins are located in the cells of the skin, inside the vacuole (Figure 2B). To extract the anthocyanins and to keep enough color (not only in red winemaking, but also in red juice production), it is necessary to disaggregate the cell wall polysaccharides, mainly the pectins (Figure 2C). In conventional winemaking, depolymerization of the cell wall and separation of polysaccharide fibers is achieved during maceration by means of soaking, fermentation temperature and mechanical treatments (i.e., punch downs, pump overs, délestage) [18]. 

In addition, cryomacerations (cold soak) by heat exchanger cooling or dry ice can be used to preferentially extract anthocyanins and aroma compounds in the absence of fermentation [19]. This is advantageous because it reduces the extraction of tannins whose solubility is lower in the absence of alcohol and better reduces astringency in young wines and juices. Another powerful technology to quickly degrade cell wall pectins and promote the extraction of anthocyanins, tannins and aroma compounds is the use of pectolytic enzymes [20], especially endo-polygalacturonases that break the pectin sequence depolymerizing the cell wall and releasing the pigments in the juice.

Currently emerging non-thermal technologies are increasingly being used to improve the extraction of bioactive compounds, such as anthocyanins, from food products, but also to decrease or eliminate spoilage or pathogenic microorganisms and sometimes even to inactivate oxidative enzymes [21,22]. Among them, continuous and discontinuous high-pressure technologies (i.e., High Hydrostatic Pressure (HHP) and Ultra High Pressure Homogenization (UHPH) [23,24,25], Pulsed Electric Fields (PEFs) [26,27,28,29], Ultrasound (US) [30,31,32,33,34] and β-irradiation [35,36,37].

This review is focused on the features of emerging non-thermal technologies that make them suitable for the extraction of anthocyanins from grape skins while protecting their natural coloring and antioxidant properties.

## 2. Use of High-Pressure Technologies to Extract Anthocyanins

The use of high-pressure technologies is growing exponentially in the food industry. A PubMed search using the keywords high, pressure and food yields 40,077 research articles in the period 1970–2021 with 36,920 since 2000. Although several technologies can be found, research with continuous (Ultra)-High Pressure Homogenization processes (UHPH and HPH) and discontinuous High Hydrostatic Pressure (HHP) technologies stand out. All of them share gentle food processing as they are non-thermal treatments with low impact on food quality, sensory constituents and nutraceutical components [25,38,39,40,41]. HHP and UHPH technologies are industrially implemented and several brands compete in the market. In batch technologies, the leading companies are Hiperbaric (https://www.hiperbaric.com/es/ (accessed on 2 November 2021)) and Avure (https://www.jbtc.com/es/north-america/foodtech/products-and-solutions/brands/avure-technologies (accessed on 2 November 2021)). In UHPH (continuous processing), the most effective technology is the one developed by Ypsicon (https://www.ypsicon.com/ (accessed on 2 November 2021)). These technologies have specific characteristics that will be described separately below.

### 2.1. High Hydrostatic Pressure (HHP)

HHP involves the application of high pressures to the food by means of a fluid (hydrostatic), which is usually water. The fluid is pumped into a high-strength steel vessel containing the food product, where pressures above 100 MPa, commonly in the range of 400–600 MPa, are reached during processing [42]. HHP treatments consist of pressurizing the food product in this pressure range for 2–10 min. The main effect of this is the destruction of the cell walls and membranes of microorganisms, but plant and animal tissue cells are also similarly affected. HHP can be considered a non-thermal technology because, even when compression produces adiabatic heating, this is quite moderate and ranges between 2 and 3 °C/100 MPa. This slight increase in temperature can be controlled by cooling the vessel or lowering the temperature of the food at the inlet. Moreover, HHP processing is not able to affect covalent bonds, so pigments, aroma and flavors are usually protected [24,38,42].

**Figure 3:**
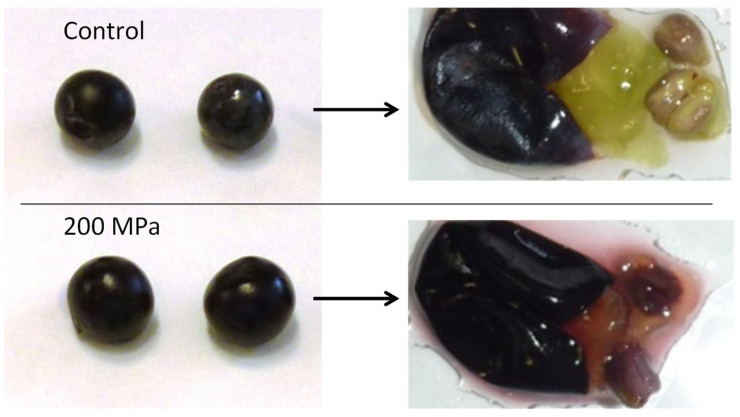
External shape and appearance of control and pressurized grapes (200 MPa, 10 min), and details of the internal structure showing colored pulp and seeds in HHP-processed grapes.

The effect of HHP on plant tissues is to damage the integrity of the cell walls, resulting in small pores or fissures that can facilitate the extraction of metabolites from the cell wall. HHP has been used to enhance the extraction of anthocyanins from grapes [23,24] and grape pomace [26]. The extraction of anthocyanins by HHP has been increased compared to controls in the range of 23–82% (Table 2). The extraction of phenolic compounds (tannins) is also increased in grapes, with a total polyphenol index of +26% [24], and the antioxidant activity of the extracts is higher than in controls [23,26]. HHP is a powerful technology to extract anthocyanins from plant tissues, and specifically from grape skin, preserving or enhancing the antioxidant capacity of the extracts, working at low temperatures (<30 °C, at 550 MPa for 10 min [24]), even under refrigeration. Furthermore, anthocyanin extraction has been reported to be selective depending on the acylation pattern and the methoxilation ratio on the B ring [23]. In grapes, anthocyanin migration from the skin to the pulp and seeds is observed after HHP treatment (Figure 3), which is evidence of cell wall poration and anthocyanin migration into the berry under the effect of pressure [24]. HHP causes the intensification of mass transfer phenomena, thus affecting cell permeability and molecule diffusion [43]. However, the external shape and structure of the berry are completely preserved (Figure 3). It is noticeable that similar extractions can be achieved in the range of 200–550 MPa, so it is possible to use milder HHP conditions, making the process cheaper while working at lower temperatures by adiabatic compression. Lower temperatures help to protect the anthocyanins during extraction and later in the ongoing process [44], probably reducing the risk of oxidation that can occur under thermal conditions.

The processing of grapes by HHP can be done with whole grapes (Figure 3), but it is also possible to process the separated skins that can be obtained from by-products such as pomace. Additionally, the stability of anthocyanins can be improved by using additives such as ethanol or other preservatives. HHP processing of berries also helps to sanitize them by easily removing yeasts and highly reducing bacterial loads [21,24]. This helps to obtain healthier anthocyanin extracts with reduced microbial loads, which facilitates the implantation of starters if these extracts are subsequently used in fermented foods [45]. Additionally, gentle extraction together with inactivation of microorganisms and higher antioxidant activity can reduce the use of antioxidants such as sulfites in the extracts, and also in the subsequent use of these extracts in food products [21,46].

**Table 2:** Emerging non-thermal technologies, processing conditions, and effects on the extraction of anthocyanin.

Emerging Non-Thermal Technology	Processing Mode	Product/Conditions	Effect	Extraction of Anthocyanins	Reference
HHP	Discontinuous	Grape skins 70 °C, 600 MPa	↑extraction ↑antioxidant activity	+23%	[23]
		Grapes <30 °C, 200–550 MPa, 10 min	↑extraction Migration of anthocyanins to pulp and seeds	+80%	[24]
		Grape by-products 70 °C, 600 MPa	↑extraction Phenols +50% ↑antioxidant activity ×3	+41%	[26]
UHPH	Continuous	Grape juice 300 MPa, 78 °C, <0.2 s	↑extraction ↑antioxidant activity	+2.6%	[47]
PEF	Continuous	Grapes 3 kV/cm, 50 pulses	↑extraction ↑juice yield +5%	×3	[48]
	Discontinuous	Grape by-products Exponential decay pulses, 70 °C, 30 kV/cm, 10 kJ/kg, 30 pulses, 2 Hz, 15 s	↑extraction Phenols +50%↑antioxidant activity ×4	+77%	[26]
	Discontinuous	Mazuelo grapes Exponential decay pulses, <30 °C, 2, 5 and 10 kV/cm, 0.4, 1.8, and 6.7 kJ/kg, 50 pulses, 1 Hz	↑extraction Phenols +20−31%	+20.3, 28.6 and 41.8% after 120 h	[49]
	Discontinuous	Pinot noir grapes Square wave bipolar pulses, 1.5 kV/cm, 15 and 70 kJ/kg, 50 Hz, pulse width of 20 μs, pulse numbers of 243 and 1033	↑extraction ↑Phenols ↑bioprotective capacity	+43−74% after 2 days	[50]
	Continuous	Cabernet sauvignon grapes Square pulses, width of 3 μs, collinear chamber, 2, 5 and 7 kV/cm; 0.56, 3.67, 6.76 kJ/kg, <23 °C, 50 pulses, 122 Hz Flow 118 kg/h. Average residence time 0.41 s	↑extraction Phenols +14−36%	+18−45% after 24 h	[51]
	Continuous	Garnacha grapes Square pulses, width of 3 μs, collinear chamber, 4.3 kV/cm, 60 μs Flow 1900 kg/h. Average residence time 0.41 s	↑extraction Phenols +23%	+25% after 7 days	[52]
	Continuous	Merlot grapes >30 kV/cm, 4.7–49.4 kJ/L Flow 500 kg/h	↑extraction ↑Phenols +23−162% Shortening of cold macerations	+17−636% after 24 h	[53]
	Continuous	Rondinella grapes Square-wave pulses at 1.5 kV/cm, 1, 5 and 10 μs, 2, 10 and 20 kJ/kg, 400 Hz Flow 250 L/h	↑extraction ↑Phenols +37%	+30% color intensity after fermentation	[54]
	Continuous	Grenache grapes Pulses of 5 kV/cm, 63.4 kJ/kg. 1800 µS (45 pulses of 40 µS) Average residence time 0.38 s. Temperature < 32 ± 2 Flow 120 kg/h	↑extraction ↑Phenols ×1.6	×2.2 after 24 h	[55]
Ultrasounds	Discontinuous	Tannat grape pomace. US bath: 15−60 °C, 0−100 W, 5−50 min	↑extraction ↑Phenols +50%	+50%	[56]
	Discontinuous	Red grape pomace US bath: 25 °C, 160 W, 40 kHz, 30 min, 0.4 W/mL, 720 J/mL	↑extraction	+59% after 5 min	[33]
	Discontinuous	Monastrell grapes US bath: 18 °C, 40 kHz, 280 W, 90 min	↑extraction ↑Phenols +9%	+8% first day of maceration	[57]
	Discontinuous	Wine lees Sonifier Cell Disruptor Model 450, high gain horn of ¾″ of diameter. Time of sonication 30–90 s	≈extraction Lower time	33% of the control time	[58]
	Discontinuous	Grape pomace Moldova variety ultrasonic transducer coupled with a function generator	↑extraction	+18% from 12.5 to 25 kHz	[59]
β-irradiation	Continuous	Tempranillo grapes 10-MeV, 50-kW Rhodotron accelerator, scan frequency of 100 Hz oses of 0 (control), 0.5, 1 and 10 kGy	↑extraction	+71% at 10 kGy	[37]

### 2.2. Ultra High Pressure Homogenization (UHPH)

UHPH consists of the continuous pressurization of a fluid to 200 MPa or more, through a special valve, and its subsequent release at low pressure (usually atmospheric pressure) [25,41,60,61]. Typical processing conditions are the use of 300 MPa with valve residence times less than 0.2 s. The process can be heat-assisted by using upstream heat exchangers, which greatly increases efficiency. The short processing time, even when high temperatures are used in the valve, produces a very gentle treatment with high nutritional and sensory quality [25]. The preservation of antioxidant activity [62], the control of oxidative enzymes such as polyphenol oxidases (PPOs) [62,63,64], the preservation of delicate aromatic molecules such as terpenes [64], and the absence of thermal markers have been observed in the processing of grape juices by UHPH [64].

**Figure 4:**
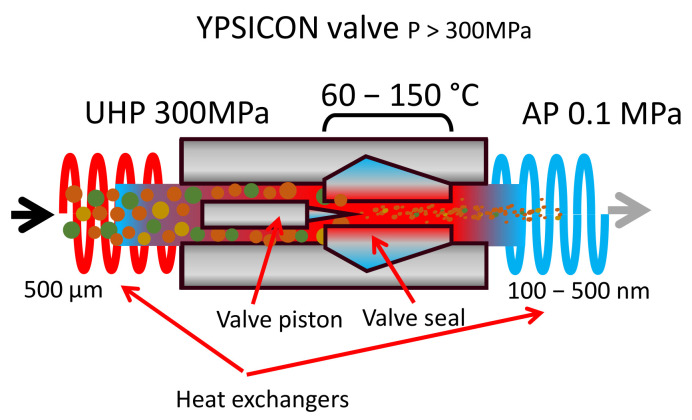
Scheme of the structure and components of a UHPH-Ypsicon valve. Intense impact and shear stresses together with the help of heating produce: pasteurization/sterilization, nano-fragmentation, enzyme inactivation, nano-coating and nano-encapsulation.

The UHPH process and the passing through the valve produce high impact forces and intense shear stresses, and the result is a significant nanofragmentation of plant tissues with removal of microorganisms, including spores depending on the temperature in the valve, inactivation of enzymes and nanofragmentation of colloidal particles. The consequence is increased extraction by cell disruption and improved bioaccessibility [65]. The mechanical effect is highly dependent on the valve design, and the antimicrobial effect with mild impact on the residence time and the design of the heat exchangers upstream and downstream of the valve [25] (Figure 4). One of the most effective designs is the one developed by Ypsicon [66].

**Figure 5:**
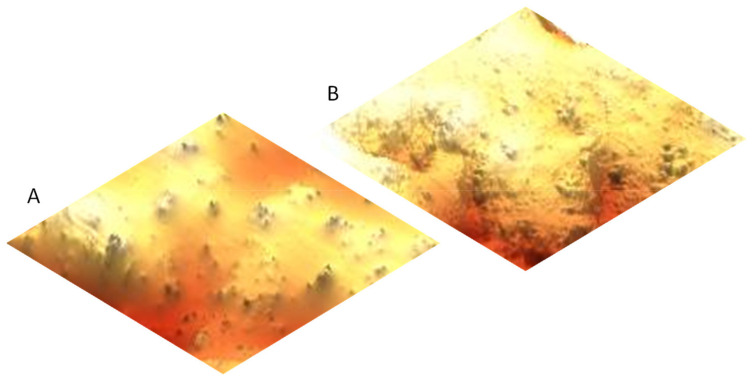
(**A**) Atomic Force Microscopy (AFM) topography of the surface of a dried red grape juice showing polyhedral granules, which are the colloidal particles of the juice (i.e., plant cell fragments and fibers). (**B**) The same dried red grape juice by AFM after UHPH treatment, with smaller granules and a flatter surface (no large polyhedral granules).

There is a size requirement concerning the maximum size of colloidal particles in the grape juice before pressurization due to the cross-sections in the fluidic components of the pump and valve. Particles in the fluid should be less than 500 µm to avoid clogging (Figure 4) [25]. After the treatment, the particles are fragmented in the range of 100–500 nm [25,64]. When grape juice, which has many colloidal constituents with a polyhedral appearance (Figure 5A), is processed by UHPH, a finer structure can be observed (Figure 5B) without large fragments [47].

## 3. Pulsed Electric Fields (PEFs) in the Extraction of Anthocyanins

**Figure 6:**
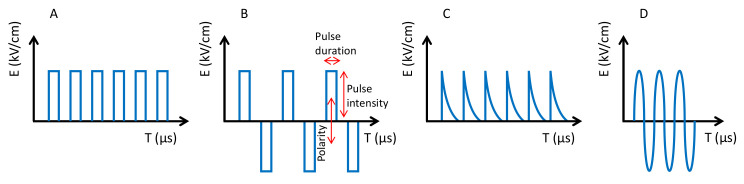
Types of pulses. (**A**) Squared monopolar. (**B**) Squared bipolar. (**C**) Exponentially decaying. (**D**) Sinusoidal.

Like the previous ones (HHP and UHPH), PEFs have become a global technology with numerous applications in food processing, preservation and stabilization [67,68,69,70,71]. PEF is based on the use of high intensity electric fields (3–40 kV/cm) for a very short time (milli-micro seconds). Food is processed by PEF when it passes through two electrodes. The Electric Field Strength (E) is the voltage (kV) divided by the distance between the electrodes (cm), i.e., E = V/d. PEF systems are currently available on an industrial scale for food processing in the range of 50–10,000 L/h for fluids and 1–70 tonnes/h for solids such as French fries. The effect of PEFs is the poration of cells at the nanoscale, which affects the selective permeability [72]. These pores are difficult to observe by electronic microscopy. However, the pores produce various effects depending on size and number, tending to increase cell permeability, thus facilitating the extraction of cell compounds (e.g., anthocyanins and many others), the entry of compounds and the temporal or definitive inactivation of cells depending on the intensity [73]. Pulses can be applied in several modalities. The main parameters are the pulse shape (i.e., squared, exponential, sinusoidal), the polarity (i.e., monopolar or bipolar), the number of pulses and the pulse duration (Figure 6). The intensity and effectiveness of the treatments depend on the above parameters with squared bipolar pulses being more effective and the number of pulses making the process more powerful. Even when the pulse duration also improves the efficacy, it should be kept at a low value because it affects the temperature of the food by ohmic heating.

**Figure 7:**
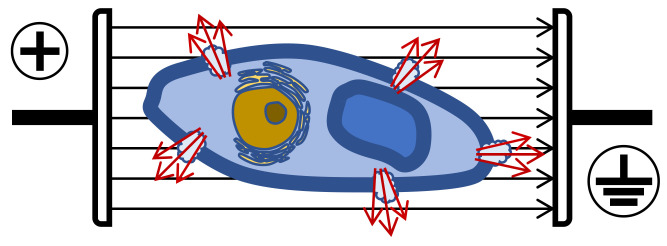
Electroporation and cell permeabilization.

Plant cells need lower intensities than microorganisms, especially bacteria, depending on size and shape. To induce permeabilization in plant cells (size 40–200 µm), E must be 1–2 kV/cm, while in microorganisms (size 1–10 µm), 12–20 kV/cm are required [74]. Therefore, to extract bioactive molecules from vegetal tissues, less than 5 kV/cm is necessary, however, for microbial inactivation, E should normally be higher than 10 kV/cm. When plant cells are pored (i.e., grape skins), the consequence is an increased extraction of biomolecules such as anthocyanins, tannins and aroma compounds (Figure 7).

At pilot and industrial scale, several works have demonstrated the efficiency of PEFs to increase the extraction of anthocyanins, and other phenols at low temperature while preserving their antioxidant capacity (Table 1). Currently grapes or by-products (grape pomace) can be processed continuously at a flow rate of several hundreds to a few tonnes of kg per hour (118 kg/h, [51]; 500 kg/h [53], 1900 kg/h [52]). Usually, the crushed gape is pumped by a progressive cavity pump [51] or a peristaltic pump [52] and later processed in a collinear chamber by applying exponentially decay pulses or, more frequently, squared pulses of an electric field strength ranging from 2 to 10 kV/cm [26,28,48,49,52,53]. Anthocyanin extraction increases in the range of 17–100% (Table 1) depending on processing conditions and post-maceration time. The temperature is increased by only 2–15 °C [55], therefore it is easy to work at room temperature or under refrigerated conditions. In addition to improved anthocyanin and phenol extraction, PEFs can be used for gentle non-thermal pasteurization of the must, thus improving the implantation of non-*Saccharomyces* starters [55] and potentially reducing the use of SO_2_. The effect of PEFs on the extraction of phenolic compounds from seeds has also been reported and should be considered in winemaking processes [75,76].

## 4. Ultrasounds (USs) in the Extraction of Anthocyanins

**Figure 8:**
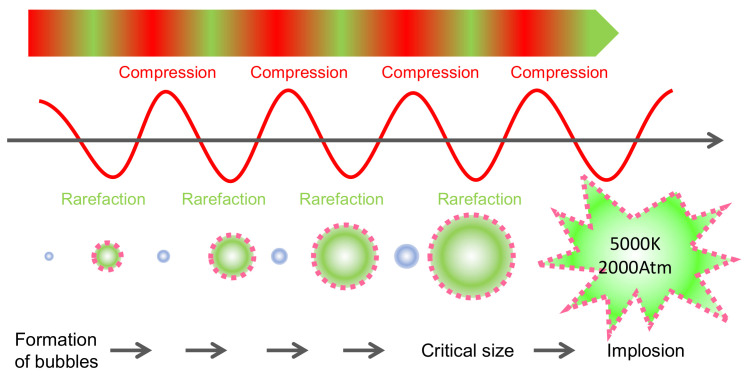
Implosion of bubbles and cavitation produced by alternative compression-rarefaction effects generated by US waves [22].

**Figure 9:**
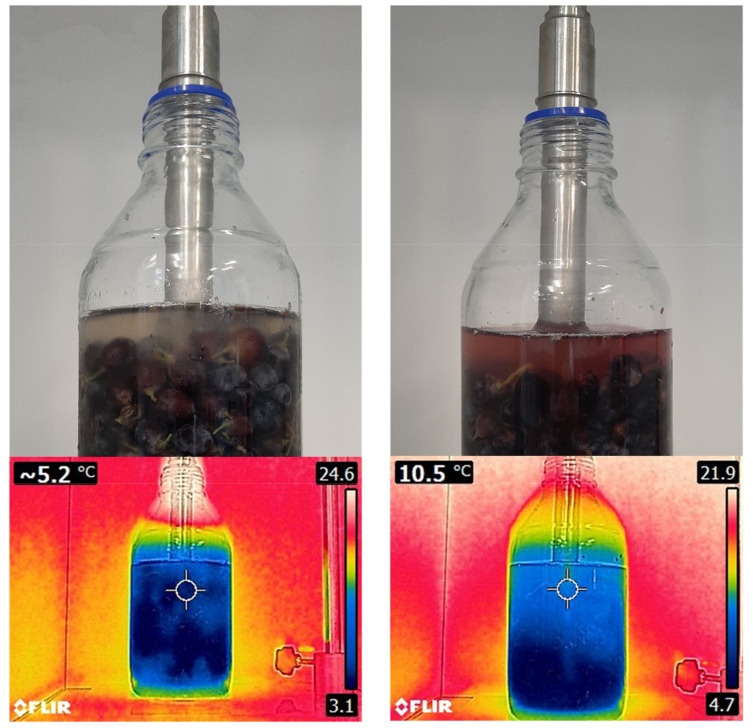
Use of USs in the extraction of grape anthocyanins and effect on temperature measured with an infrared camera. Left: before ultrasonication, right: after US treatment.

Ultrasounds (USs) are mechanic waves with a frequency above 20 kHz, which is not perceptible to the human ear (typically in the range 20 Hz–20 kHz) [77]. It is a key technology for obtaining bioactive compounds (e.g., anthocyanins), like the others described above, as it can be considered a sustainable ‘green’ extraction method [78] as it does not use organic solvents and is gentle to heat-sensitive molecules [79]. The compression and rarefaction of the products produced by the US waves produce the successive reduction in size and expansion of the bubbles formed by cavitation (Figure 8). When these bubbles collapse, large amounts of energy are released, reaching localized temperatures of 5000 °K and pressures of 200 MPa [80]. These phenomena are responsible for the depolymerization of biostructures [80] and facilitate the extraction of molecules from plant tissues. Depolymerization can occur by bubble collapse, cavitation or degradation of the polymer by impact with radicals formed during sonication [81]. Depolymerization of cell wall polysaccharides accelerates the release of anthocyanins from the skin cells in grapes (Figure 9). The extraction of anthocyanins in water within a few minutes and the increase in temperature due to the cavitation effect can be observed. High power ultrasounds with the best extraction potential are considered to be in the range of 20–25 kHz [78].

Figure 9 shows the application of USs on grape berries by means of a sonotrode and reveals, after a few minutes, how the anthocyanins are extracted to the surrounding media (water) due to the depolymerization of the cell walls of the grape skins. Additionally, the heating effect produced by cavitation can be observed, which in this case is about 5 °C in the center of the flask according to infrared thermography.

**Figure 10:**
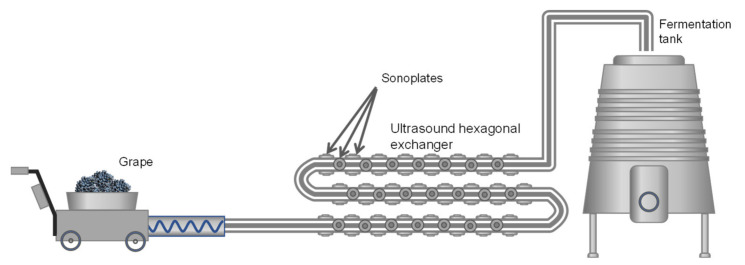
Cavitation cells arranged in a hexagonal tubular exchanger with the sonoplates for applying US waves [22].

There are several systems for applying USs to plant tissues with the aim of favoring the extraction of compounds: Ultrasound baths, sonotrodes, sonoplates. However, on an industrial scale, the most effective system is the use of continuous tubular exchangers on the external surface of which sonoplates are distributed to apply US waves during the flow of the mash or liquid through the exchanger. For a better distribution of the sonoplates on the exchange surface, the section is usually hexagonal instead of circular (Figure 10).

This technology has been used to process Tempranillo grapes, achieving the same anthocyanin content in a final wine after only continuous US treatment and 72 h of skin maceration as in the control wine [82]. In discontinuous treatment at the laboratory scale, the USs have been shown to increase the extraction of anthocyanins and phenols by more than 50% compared to controls [56]. US can also be applied continuously after the application of pectolytic enzymes at industrial level, increasing color intensity by 18% and total polyphenols by 21% in wines [57]. The use of US-assisted extraction can be improved by optimizing other physicochemical parameters (temperature, ethanol and time), thus reaching a maximum of 6.26 mg/mL under the best conditions of 45.14 °C, 52.3% ethanol and 24.5 min [83]. USs can be used to improve extraction and/or reduce extraction time in grapes [56,57,82], and by-products such as pomace [33] and lees [58]. USs have been applied to *Vitis vinifera* L. varieties Cabernet Franc [84], Tempranillo [82], Tannat [56], and Monastrell [57]. The influence of the US frequency has also been analyzed, considering the values of 12.5, 25, and 37.5 kHz, as well as from 12.5 to 25 kHz, the extraction of anthocyanins increased by 18% in grape pomace, however, the higher the frequency, the lower the extraction [59].

## 5. Effect of E-Beam Irradiation in the Extraction of Anthocyanins

**Figure 11:**
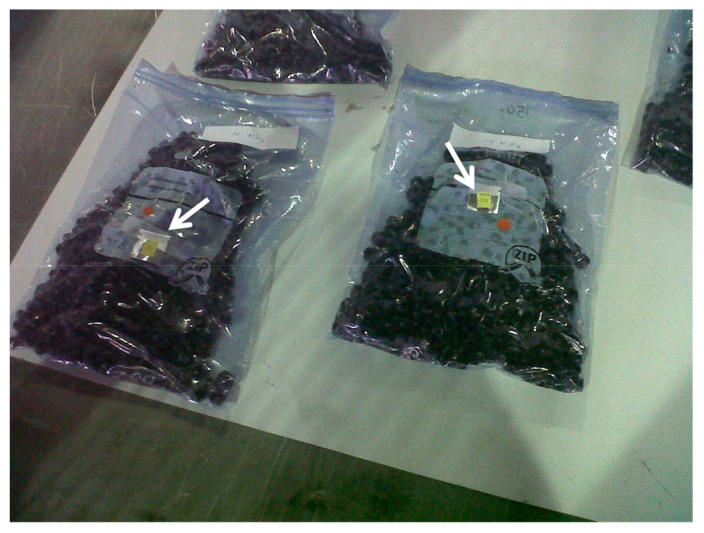
Red grapes in plastic bags after e-beam irradiation. The white arrows indicate the location of radiochromic dosimeters.

Electron beam (e-beam) irradiation or β-irradiation involves the use of accelerated electrons at high energy, typically 10 MeV [21,37,85], to process foods and eliminate microorganisms, allowing pasteurization (1–5 kGy) or sterilization (>10 kGy) depending on the dose [21,37,85]. The irradiation dose is measured in Grays (Gy) or kGrays (kGy). One Gy is defined as the absorption of 1 Joule of energy per kg of irradiated mass. Irradiation is widely used to preserve food in more than 55 countries and is considered a safe technology approved by WHO, FAO and IAEA [86]. This technique is cheap on a large scale, environmentally friendly and time efficient [87]. e-Beam irradiation is a complex technology that requires expensive irradiation accelerators and large-scale facilities. e-Beam irradiation can be applied on an industrial scale in a continuous process [21]. The radiation dose can be monitored and verified by placing radiochromic dosimeters on the treated food (Figure 11). This can be used to verify the real dose received by the food at every width. e-Beam irradiation can be considered a gentle non-thermal technology with temperature increments of less than 5 °C at doses up to 10 kGy [21]. In addition, e-beam irradiation has been proposed as an alternative to sulphites in wine preservation [88] and has demonstrated its ability to delay browning in plant foods [89]. However, some negative effects have been observed such as loss of aroma [37] and reduction in vitamin C content [85], due to free radical-mediated oxidation [90].

**Figure 12:**
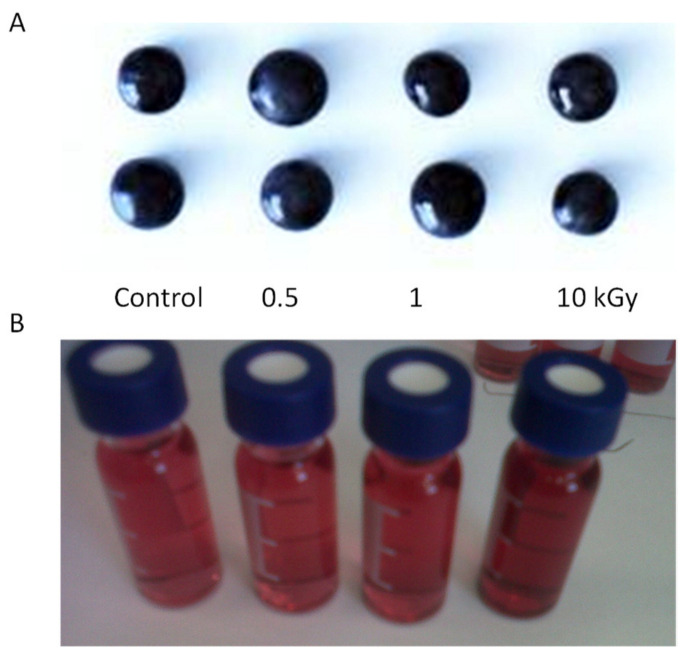
Effect of e-beam irradiation on the external appearance of grapes (**A**). Running juice from grapes processed by e-beam irradiation at various doses (**B**).

Even when the external appearance of the grapes after irradiation remains unchanged (Figure 12A), the release of some juice in the bags can be observed, especially at high doses (10 kGy). This leakage of juice from the grapes shows the weakening of the plant tissues due to irradiation. The main effect of e-beam irradiation on plant tissues is the fragmentation of fibrillar polymers such as pectins and other polysaccharides, promoting the release and extraction of cell components, including anthocyanins. It has been reported that the molecular weight of pectins can be reduced by 90% using doses of 3–10 kGy [91]. The effect on grapes is the increased extraction of phenols and anthocyanins [37], which can be observed in the more intense color of the running juice, especially when grapes are processed at 10 kGy (Figure 12B). Up to 1 kGy, the anthocyanin extraction is low and not too high compared to the controls, however at 10 kGy, the anthoyanins extracted in the running juice were 125 mg/L compared with 72 mg/L on average in the controls (+71%) [37]. Better antioxidant and sensory properties and higher phenol content have also been observed in grapes processed up to 2 kGy [92]. With blueberries, the use of e-beam irradiation at doses below 3 kGy has demonstrated to be a gentle processing that does not affect monomeric anthocyanin and antioxidant activity [93]. Protection of anthocyanins, color, phenols and antioxidant activity has also been observed in strawberries processed at 1 kGy [94].

## 6. Pulsed Light 

Pulsed light (PL) treatment involves the application of high-intensity, low-duration pulses of radiation in the 200 nm (UV) to 2500 nm (IR) range [95,96]. The intensity can be higher than 10^6^ fold that of sunlight at sea level in the summer midday and the duration ranges from micro to milliseconds. Detailed parameters for pulsed light processing conditions have recently been revised [97]. The temperature rise after standard PL treatments is usually less than 5 °C, so it can be considered a mild non-thermal technology that can be used in delicate foods [96].

The ability to extract anthocyanins and phenols from plant tissues is lower than some of the previous techniques and the literature reports inconclusive results. Non-significant differences have been found in the anthocyanin content of wines made from PL-processed grapes compared to controls [98]. Temperature increases in grape skins of 2–3 °C after pulsed light treatments have also been reported without severe damage to the skin surface observed by AFM scanning [99]. Furthermore, PL processing of strawberries at 4–8 J/cm^2^ does not affect the quality and antioxidant capacity [100].

## 7. Conclusions

The use of emerging non-thermal technologies is a successful tool for the extraction of anthocyanins from grapes, increasing the yield, accelerating the process and preserving the antioxidant capacity. Many of these techniques can be applied in continuous flow (UHPH, PEF, US, Irradiation and PL), which is suitable for industrial processing. Most of these techniques can work at room temperature or even using refrigerated crushed grapes, although temperature is always a synergistic parameter. These technologies can be used for the extraction of anthocyanins from grapes, and also from by-products such as pomace, generating high-value pigments from them. Additionally, emerging technologies can be used to improve the winemaking process by increasing the extraction of anthocyanins and phenolic compounds in maceration and controlling oxidations.

## Figures and Tables

**Table 1:** Molecular structure, substitution pattern in the B-ring (R1 and R2), acylation patterns (R3), maximum λ (nm), and color of grape’s anthocyanins.

Anthocyanin	R1	R2	R3	λ max ^1^	Colour	[M]^+^/Aglycon (*m/z*)^2^
Delphinidin-3-*O*-gucoside	‒OH	‒OH	‒H	526.9	Red	465/303
Cyanidin-3-*O*-glucoside	‒OH	‒H	‒H	518.4	Orange-red	449/287
Petunidin-3-*O*-glucoside	‒OH	‒OCH_3_	‒H	528.1	Red	479/317
Peonidin-3-*O*-glucoside	‒OCH_3_	‒H	‒H	518.4	Orange-red	463/301
Malvidi-3-*O*-glucoside	‒OCH_3_	‒OCH_3_	‒H	529.3	Red	493/331
Delphinidin-3-*O*-(6-*O*-acetyl)-glucoside	‒OH	‒OH	‒COCH_3_	529.3	Red	507/303
Cyanidin-3-*O*-(-6-*O*-acetyl)-glucoside	‒OH	‒H	‒COCH_3_	520.8	Red	491/287
Petunidin-3-*O*-(-6-*O*-acetyl)-glucoside	‒OH	‒OCH_3_	‒COCH_3_	530.5	Bluish-red	521/317
Peonidin-3-*O*-(-6-*O*-acetyl)-glucoside	‒OCH_3_	‒H	‒COCH_3_	520.8	Red	505/301
Malvidin-3-*O*-(-6-*O*-acetyl)-glucoside	‒OCH_3_	‒OCH_3_	‒COCH_3_	530.5	Bluish-red	535/331
Delphinidin-3-*O*-(6-*O*-*p*-coumaroyl)-glucoside	‒OH	‒OH	‒COCH = CHC_6_H_4_‒OH	534.2	Bluish-red	611/303
Cyanidin-3-*O*-(-6-*O*-*p*-coumaroyl)-glucoside	‒OH	‒H	‒COCH = CHC_6_H_4_‒OH	525.7	Red	595/287
Petunidin-3-*O*-(-6-*O*-*p*-coumaroyl)-glucoside	‒OH	‒OCH_3_	‒COCH = CHC_6_H_4_‒OH	535.4	Bluish-red	625/317
Peonidin-3-*O*-(-6-*O*-*p*-coumaroyl)-glucoside	‒OCH_3_	‒H	‒COCH = CHC_6_H_4_‒OH	524.5	Red	609/301
Malvidin-3-*O*-(-6-*O*-*p*-coumaroyl)-glucoside	‒OCH_3_	‒OCH_3_	‒COCH = CHC_6_H_4_‒OH	535.4	Bluish-red	639/331
Malvidin-3-*O*-(-6-*O*-caffeoyl)-glucoside	‒OCH_3_	‒OCH_3_	‒COCH = CHC_6_H_3_‒(OH)_2_	536.6	Bluish-red	655/331
Molecular structure			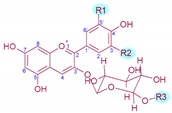			

^1^ Obtained experimentally with HPLC-DAD-ESI/MS; ^2^ From [17].
